# Deep Learning With 18F-Fluorodeoxyglucose-PET Gives Valid Diagnoses for the Uncertain Cases in Memory Impairment of Alzheimer’s Disease

**DOI:** 10.3389/fnagi.2021.764272

**Published:** 2021-12-15

**Authors:** Wei Zhang, Tianhao Zhang, Tingting Pan, Shilun Zhao, Binbin Nie, Hua Liu, Baoci Shan, Alzheimer’s Disease Neuroimaging Initiative

**Affiliations:** ^1^Beijing Engineering Research Center of Radiographic Techniques and Equipment, Institute of High Energy Physics, Chinese Academy of Sciences, Beijing, China; ^2^School of Nuclear Science and Technology, University of Chinese Academy of Sciences, Beijing, China; ^3^School of Physics and Microelectronics, Zhengzhou University, Zhengzhou, China

**Keywords:** Alzheimer’s disease, deep learning, FDG-PET, uncertain, neuropsychologial assessment

## Abstract

**Objectives:** Neuropsychological tests are an important basis for the memory impairment diagnosis in Alzheimer’s disease (AD). However, multiple memory tests might be conflicting within-subjects and lead to uncertain diagnoses in some cases. This study proposed a framework to diagnose the uncertain cases of memory impairment.

**Methods:** We collected 2,386 samples including AD, mild cognitive impairment (MCI), and cognitive normal (CN) using 18F-fluorodeoxyglucose positron emission tomography (FDG-PET) and three different neuropsychological tests (Mini-Mental State Examination, Alzheimer’s Disease Assessment Scale-Cognitive Subscale, and Clinical Dementia Rating) from the Alzheimer’s Disease Neuroimaging Initiative (ADNI). A deep learning (DL) framework using FDG-PET was proposed to diagnose uncertain memory impairment cases that were conflicting between tests. Subsequent ANOVA, chi-squared, and *t*-test were used to explain the potential causes of uncertain cases.

**Results:** For certain cases in the testing set, the proposed DL framework outperformed other methods with 95.65% accuracy. For the uncertain cases, its positive diagnoses had a significant (*p* < 0.001) worse decline in memory function than negative diagnoses in a longitudinal study of 40 months on average. In the memory-impaired group, uncertain cases were mainly explained by an AD metabolism pattern but mild in extent (*p* < 0.05). In the healthy group, uncertain cases were mainly explained by a non-energetic mental state (*p* < 0.001) measured using a global deterioration scale (GDS), with a significant depression-related metabolism pattern detected (*p* < 0.05).

**Conclusion:** A DL framework for diagnosing uncertain cases of memory impairment is proposed. Proved by longitudinal tracing of its diagnoses, it showed clinical validity and had application potential. Its valid diagnoses also provided evidence and explanation of uncertain cases based on the neurodegeneration and depression mental state.

## Introduction

Neuropsychological tests, such as Mini-Mental State Examination (MMSE) (Folstein et al., 1975), Alzheimer’s Disease Assessment Scale-Cognitive Subscale (ADAS-Cog) (Mohs et al., 1983), and Clinical Dementia Rating (CDR) (Morris, 1993), are common methods that evaluate cognitive performance and also play a key role in screening for dementia (Creavin et al., 2016). Among the different aspects of cognition, memory impairment is considered the most primary and common cognitive impairment both in the progress of mild cognitive impairment (MCI) and in Alzheimer’s disease (AD) (Gauthier et al., 2006; Wilson et al., 2011; Scheltens et al., 2016). However, doubt on their reliability and validity exists (Rikkert et al., 2011; Spencer et al., 2013; Jiang et al., 2020), and the conflict between test results within subjects can be severe, especially in uncertain dementia cases (Perneczky et al., 2006; Trzepacz et al., 2015). This can lead to poor and uncertain outcomes of dementia diagnoses (Matthews et al., 2008), which can be unstable compared with neuropathologic results from MRI or PET scans (Shim et al., 2013).

Moreover, the cause of these uncertain cases remains unclear. Major explanations include the lack of sensitivity of these tests (Wind et al., 1997; De Jager et al., 2002; Perneczky et al., 2006), especially in diagnosing between cognitive normal (CN) and MCI (Mitchell, 2009), and different evaluation methods and focus between tests (Trzepacz et al., 2015; Bergeron et al., 2017). However, few studies focused on the neurological state and studied the brain images of these subjects to get a convincing explanation of uncertain cases. A more stable and reliable diagnosis method is needed for uncertain cases, and more explanation and evidence are also needed to help understand and overcome these uncertain cases (Gaugler et al., 2013).

Inspired by these works, to explore a diagnosing method and an explanation with evidence for the uncertain cases in memory impairment related to AD, we tried to diagnose the uncertain cases using a designed deep learning (DL) framework on 18F-fluorodeoxyglucose positron emission tomography (FDG-PET) using a large set of samples, verify its validity using longitudinal memory function progress, and figure out neurological evidence and cause using groupwise statistical analyses.

## Related Works

In the field of computer-aided diagnosis (CAD), more researchers are focusing on analyzing neuroimages using the DL algorithm (LeCun et al., 2015). It abstractly extracts high-dimensional features along with a powerful classification ability and does not rely on expert-designed features such as traditional methods (e.g., linear regression and support vector machine). For diagnosing AD and related pathology, the neurodegeneration revealed by FDG-PET hypometabolism and atrophy on MRI are both defined as multimodal biomarkers (Jack et al., 2018; Zhang et al., 2020; Wang et al., 2021). The diagnosis (Ortiz et al., 2016) or prediction (Shen et al., 2019; Spasov et al., 2019) based on deep neural networks was proposed and showed high accuracy with fast implementation.

However, most CAD researches dealt with certain labeled samples (Shen et al., 2017) before training or testing the performance of models, but the classification potential of DL on judging the uncertain and unlabeled samples should be more exploited. As previous works (Hosokawa et al., 2015; Son et al., 2020) started to use the patch-based 2D convolutional neural network (CNN) to distinguish uncertain β-amyloid PET and achieved the level for clinical usage, this network implementation might not be suitable for detecting lesions in the images of uncertain memory-impaired cases because of the unknown lesion location for making patches and potential loss of texture information between 2D layers.

Inspired by these studies, we proposed a semi-supervised learning framework based on 3D CNN (Du et al., 2015) that extracts discriminative features using certain impaired samples and provides guiding diagnoses for the uncertain impaired samples. Moreover, to optimize the network for PET-FDG-based diagnosis, several important network designs were implemented. First, we replaced the original stacked fully connected layer with a 1 × 1 × 1 convolution layer, inspired by the former study (Lin et al., 2015). In practice, this largely simplified the network while still keeping high performance and was able to train large 3D PET images sized 96 × 96 × 96 and, therefore, better preserving the texture information between axial layers in the PET image. Moreover, from a biological and pathological scope, we also gave evidence and explanation of uncertain cases based on neurodegeneration and depression mental state.

## Materials and Methods

### Study Population

All data used were obtained from the open-source project the Alzheimer’s Disease Neuroimaging Initiative (ADNI),^1^ which is the largest ongoing project for the analysis of AD, covering all subphases of ADNI project from September 2006 to October 2019 (ADNI1, ADNIGO, ADNI2, and ADNI3). All available FDG-PET and corresponding MRI images up to April 2020 were collected to ensure a large data amount. One case of data included FDG-PET/CT scanning for glucose metabolism, T1-weighted magnetization prepared rapid gradient-echo (MPRAGE) MRI, memory assessment in three major neuropsychological tests, namely, MMSE, ADAS-Cog, and CDR, and global deterioration scale (GDS, with 15 questions detailed in Supplementary Table 1) for depression mental state. All scale tests were carried out within 6 months to FDG-PET. Moreover, we expanded our baseline data by searching for all available longitudinal memory assessments that baseline subjects went through. The longitudinal time lengths are limited to 6–96 months after baseline, for a sufficient sample amount. These changes in the longitudinal study were counted every 6 months.

### Neuropsychological Tests and Grouping Criteria

Three major neuropsychological tests including MMSE, ADAS-Cog, and CDR were carried out within 6 months and the images were collected for each FDG-PET scan. We did not choose other popular tests, such as the Montreal Cognitive Assessment (MoCA), because these were less applied in ADNI set. These neuropsychological tests are comprehensive evaluations of different cognitive functions, and memory is the most significant and primary one. The delayed word recall test is applied in the same way both in MMSE and ADAS-Cog. To make different tests more comparable, this study concentrated on the delayed word recall tests of MMSE (MMSE-Recall) and ADAS-Cog (ADAS-Cog-Recall), and CDR score of memory (CDR-Memory). In detail, MMSE-Recall was scored 0–3 based on how many words of 3 were recalled, ADAS-Cog-Recall was scored 0–30 based on how many words of 30 were recalled, and CDR-Memory was scored 0, 0.5, 1, 2, and 3 as healthy, suspected, mild, moderate, and severe memory impairment.

**FIGURE 1:**
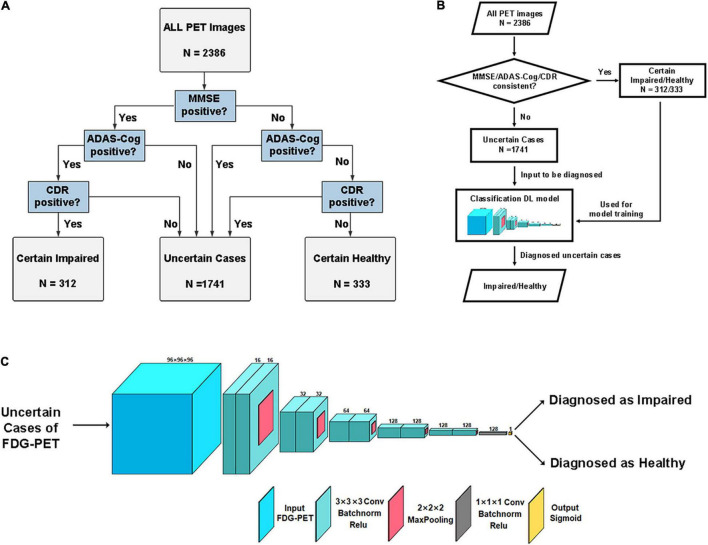
**(A)** Flowchart of data grouping criteria, using memory scores of these tests. **(B)** Flowchart of this research, including grouping, training, and utilizing of the DL model. The uncertain samples were diagnosed using this frame in the end. **(C)** The concrete structure of the designed three-dimensional CNN model. MMSE, Mini-Mental State Examination; ADAS-Cog, Alzheimer’s Disease Assessment Scale-Cognitive Subscale; CDR, Clinical Dementia Rating.

Each FDG-PET image was grouped based on whether the memory cognitive impairment was certainly impaired or healthy in the three neuropsychological tests. First, if MMSE-Recall ≤ 1, ADAS-Cog-Recall < 12 (defined by “mean − standard deviation”), and CDR-Memory ≥ 1, the case will be grouped into “certain impaired.” In reverse, if MMSE-Recall > 1, ADAS-Cog-Recall > 23 (defined by “mean + standard deviation”), and CDR-Memory = 0, the case will be grouped into “certain healthy.” The rest of the cases that the three tests are conflicting with each other will be grouped as “uncertain cases” and diagnosed using the DL framework proposed in this study. The whole grouping is shown in Figure 1A.

### Image Acquisition and Preprocessing

All raw FDG-PET and MRI images were acquired following the standardized ADNI protocols (Jack et al., 2008; Jagust et al., 2010) and processed following the same criterion: PET images were first registered to corresponding T1-weighted MPRAGE or inversion recovery-spoiled-gradient recalled echo (IR-FSPGR) MRI native space using the normalized mutual information method, then spatially normalized to the Montreal Neurological Institute (MNI) template using warping parameters derived from the individual MRI normalization performed previously *via* the routine of unified segmentation algorithm (Ashburner and Friston, 2005). Finally, images were spatially smoothed using the Gaussian kernel of 8 mm full width at half maximum to improve the signal to noise ratio and overlapped using a customized binary mask for the whole brain, all completed using Statistical Parametric Mapping 12 (SPM12).^2^ Voxel standard uptake value (SUV) was divided by mean uptake of the whole pons (Whitwell et al., 2018) to generate a standard uptake value ratio (SUVr). Because data used in this study are from a large multisite project ADNI, including a total of 59 sites, partial volume correction (Mullergartner et al., 1992) was not included to avoid adding unnecessary image variances across sites (Klunk et al., 2015) and weaken the generality of multisite data. Full information about the acquisition of data in the ADNI Laboratory of Neuroimaging (LONI) database is provided at http://adni.loni.usc.edu/data-samples/data-types/.

### Deep Learning Classification Framework

After grouping certain and uncertain cases, the corresponding FDG-PET images were used to diagnose uncertain cases using the DL framework. First, for evaluating the classification performance of each method, all images (*n* = 645) in a certain group were evaluated using fivefold cross-validation. Then, all FDG-PET images in the uncertain group were placed into the trained DL framework, which was diagnosed to be memory-impaired/healthy. The whole flowchart is shown in Figure 1B.

A specially designed convolution neural network (CNN) model was used to classify impaired/healthy from the FDG-PET image of each case. The structure of the model is shown in Figure 1C. We used a 3D convolution layer of size 3 × 3 × 3 with stride 1, followed by a batch normalization layer and a rectified linear unit (ReLU) activation layer for non-linearity. The downsampling was performed using 3D max-pooling of size 2 × 2 × 2. The number of filters was multiplied from 16 to 128 as the downsampling goes. Finally, a convolution process with 1 × 1 × 1 3D convolution was performed to summarize the high-dimensional features and ended with a dense layer with sigmoid activation as classification output. Using 3D convolution sized 1 replacing stacked fully connected layers reduced network parameters that need training from 230 million to 882,000. The model was trained using Adam (Kingma, 2015) optimizer with the learning rate of 0.001 and loss of binary cross-entropy, using a mini-batch size of 4 considering both the efficiency and RAM size. To decrease overfitting, the training process used early stopping tuning by stopping when the training loss did not significantly decline by 0.01 within 10 epochs. The whole model was accomplished using Keras 2.1.2^3^ framework on TensorFlow 1.14.0^4^ backend, with a GPU of NVDIA RTX2080Ti^5^.

### Evaluation of Performance

First, the classification performance in certain cases was tested using fivefold cross-validation. For each iteration of cross-validation, 60% of images were used as a training set, 20% of images were used as a testing set, and 20% of images were used as a validation set. To compare the performance of the proposed 3D-CNN-DL framework, we also used three layers of multilayer perceptron, 3D ResNet implemented by Hara et al. (2017), C-support vector machine (SVM), Nu-SVM (with the linear or radial kernel using LIBSVM toolbox^6^) (Chang and Lin, 2011), linear regression, and logistic regression for the classification task. The common parameters for classification, namely, accuracy, precision, sensitivity, specificity, F1 score, and area under the curve (AUC) of receiver operating characteristic curve (ROC), were used.

Second, to evaluate the accuracy of the DL framework’s diagnoses on uncertain cases, we used the memory testing scores in the progress of the longitudinal study of each subject, with 40 months on average. Because the longitudinal progress of memory impairment is a key concern of AD and also a reliable marker indicating whether true impaired or healthy state at baseline, a nice diagnosis framework should tell apart the subjects between memory impairment in progress and healthy memory function, using baseline FDG-PET as inputs.

Moreover, to interpret the high-dimensional features of the learned DL framework, two unsupervised dimension reduction methods, namely principal component analysis (PCA) and *t*-distributed stochastic neighbor embedding (t-SNE) (van der Maaten and Hinton, 2008), were applied to topologically represent and visualize the features in the last flatten layer of the DL framework to be a 2D scatter plot, where similar abstract feature vectors are spatially close to each other as scatters.

Time complexity evaluation was performed for each method. The DL algorithms were evaluated using a special metric named floating-point operations per second (FLOP) because the big “O” is not suitable for too many free variables in the DL networks. Other traditional methods were evaluated using the big “O” notation.

### Statistical Analysis

The *t*-test was applied between both voxel-wise and region of interests (ROI)-wise SUVr, and the multiple comparison correction was applied by family-wise error (FWE) or false discovery rate (FDR) depending on the extent of significance, with cluster extent > 5. The age, gender, education level, and ApoE gene types were taken into account as covariates because they could reasonably influence the neurodegeneration in FDG-PET. The *t*-test was also applied to test scores between groups.

Two-way ANOVA was applied to judge whether the variance of total GDS scores was significantly contributed by certain/uncertain, impaired/healthy, and the interaction between these two factors. Because of the different sample sizes between groups, groups will be randomly sampled to equal the smallest size 294 by applying two-way ANOVA. Considering the randomness of sampling, we repeated the random sampling and applied ANOVA 100 times, and recorded the mean *p*-value to ensure a credible significance.

The chi-squared test was applied to judge the significant ratio difference between groups, such as gender, ApoE, and each single GDS test with binary value in comparisons. *P*-values in GDS were strictly corrected by FWE because 15 tests and a total score were tested and listed.

Linear regression was applied to evaluate the longitudinal tendency of neuropsychological test scores, using months after baseline as an independent variable and scores as a dependent variable. The 95% CI of the fitted result was shown.

All statistical tests were performed using the statistics and machine learning toolbox in MATLAB R2019^7^.

## Results

### Demographic Information

In total, 2,386 samples were obtained from 1,247 subjects and tested using FDG-PET and neuropsychological tests. These samples included 332 subjects with AD, 1,347 subjects with MCI, and 707 subjects with CN. According to the grouping criteria of memory test performance, 312 subjects were grouped as “certain impaired” cases, 333 subjects were grouped as “certain healthy” cases, and 1,741 subjects were grouped as uncertain cases. All demographic information can be found in Table 1, which are grouped into certain or uncertain cases.

**TABLE 1:** Demographic information of all cases.

	Certain impaired	Certain healthy	Uncertain	*p*
N	312	333	1,741	
AD/MCI/CN	158/77/77	29/45/259	315/816/610	<0.001
Age (years)	75.68 (7.26)	74.58 (5.87)	75.65 (7.46)	0.03
Sex (F)	41.35%	52.25%	38.37%	<0.001
Education (years)	6.50 (3.40)	6.75 (3.39)	6.57 (3.42)	0.61
APOE ε4+^#^	68.27%	21.69%	45.97%	<0.001
MMSE-recall	0.19 (0.39)	2.87 (0.33)	2.02 (1.09)	<0.001
ADAS-Cog-Recall	8.49 (2.45)	25.02 (1.84)	17.54 (3.92)	<0.001
CDR	1.00 (0.49)	0.00 (0.03)	0.43 (0.28)	<0.001
CDR-memory	1.36 (0.51)	0.00 (0.00)	0.49 (0.35)	<0.001
GDS^##^	1.48 (1.54)	1.71 (1.94)	1.54 (1.84)	0.08
GDS-energy+^##^	80.27%	76.44%	69.55%	<0.001

*^#^APOE information of six samples were lacking in the dataset and not counted.*

*^##^85 GDS information were lacking and not counted. APOE ε4, apolipoprotein ε4; MMSE, Mini-Mental State Examination; ADAS-Cog, Alzheimer’s Disease Assessment Scale-Cognitive Subscale; CDR, Clinical Dementia Rating; GDS, Global Deterioration Scale.*

### The Proposed Deep Learning Framework Outperformed Others in Classifying Certain Cases

Nine different models were trained on the FDG-PET images of certain cases with fivefold cross-validation. After evaluating the binary classification performance, the DL framework has an obvious comprehensive advantage over other models, separately in accuracy (95.90%), precision (97.01%), sensitivity (93.59%), F1 score (95.27%), and AUC (98.15%) (Table 2). The timing-based evaluation was also performed and is recorded in Table 2. For three DL-based methods, the training was all within 40 epochs and 15 min. FLOP was also recorded for network time complexity. For other traditional methods, a big “O” notation of time complexity was estimated.

**TABLE 2:** Classification performance of different models in the testing set, by fivefold cross-validation.

Algorithms	Accuracy (%)	Precision (%)	Sensitivity (recall) (%)	Specificity (%)	F1 score (%)	AUC (%)	Time complexity^#^
Our proposed network	95.50	97.01	93.59	97.30	95.27	98.15	FLOP = 181 G
MLP	92.25	93.09	90.71	93.69	91.88	97.14	FLOP = 0.5 G
3D ResNet	93.18	96.53	89.10	97.00	92.67	97.59	FLOP = 93.9 G
C-SVM (linear kernel)	93.80	94.16	92.95	94.59	93.55	97.81	O(dn)
C-SVM (radial kernel)	84.81	83.23	85.90	83.78	84.54	92.31	O(dn^2^)
Nu-SVM (linear kernel)	91.16	91.53	90.06	92.19	90.79	96.05	O(dn)
Nu-SVM (radial kernel)	90.85	90.94	90.06	91.59	90.50	95.99	O(dn^2^)
Linear regression	92.56	93.42	91.03	93.99	92.21	97.65	O(dn)
logistic regression	91.16	89.97	91.99	90.39	90.97	96.30	O(dn^2^)

*DL, deep learning; MLP, multilayer perceptron; SVM, support vector machine; AUC, area under the curve; FLOP, floating-point operations per second.*

### The Proposed Deep Learning Framework Diagnosing Uncertain Cases and Proved Clinical Validity Using the Longitudinal Study

To judge whether the diagnoses classified using the DL framework in the uncertain cases are reliable, we chose to track the longitudinal memory function progress of each uncertain case and regarded it as an evaluation criterion for the diagnoses with the baseline FDG-PET.

The total longitudinal cases are 8,870 for ADAS-Cog-Recall, 8,865 for MMSE-Recall, 9,323 for CDR-Memory, and the cases that three tests completed at the same time are 6,912. The longitudinal time lengths are 40.38 ± 29.32 months for MMSE-Recall, 40.91 ± 29.68 months for ADAS-Cog-Recall, and 42.07 ± 30.40 months for CDR-Memory.

**FIGURE 2:**
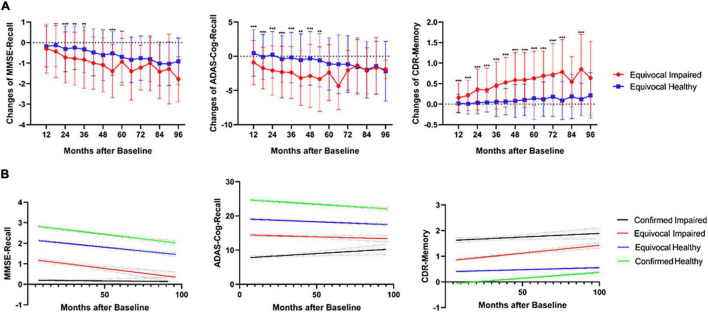
**(A)** Significantly different longitudinal changes of three neuropsychological tests (MMSE, ADAS, and CDR) between impaired and healthy uncertain cases diagnosed using the DL framework, calculated for every 6 months from 6 to 96 months. ***p < 0.001, **p < 0.01, all family-wise error (FWE) corrected. **(B)** Linear regression of longitudinal three neuropsychological tests for all four groups including the uncertain diagnoses, from 6 to 96 months. The 95% CIs were shown in gray color.

After the DL framework had diagnosed these uncertain cases, as for the longitudinal changes (Figure 2A), impaired diagnoses showed significantly more memory decline than healthy diagnoses in all 6 years for CDR-Memory and mainly in the first 5 years for the other two tests. Longitudinal cases more than 5 years are rare in amount, which might explain the insignificance after 5 years. The linear regression results also agreed with this difference (Figure 2B). For MMSE-Recall and ADAS-Cog-Recall, three groups except certain impaired group remain declining as the ages grow, the uncertain impaired group shows severe memory function in the long term (mean MMSE-Recall = 0.35 and mean ADAS-Cog-Recall = 13.35, after 96 months), which is close to certain impaired group (mean MMSE-Recall = 0.14 and mean ADAS-Cog-Recall = 10.25, after 96 months), while the uncertain healthy group shows a healthy state in the long term (mean MMSE-Recall = 1.46 and mean ADAS-Cog-Recall = 17.47, after 96 months), which is close to certain healthy group (mean MMSE-Recall = 2.03 and mean ADAS-Cog-Recall = 22.07, after 96 months). For CDR-Memory, the uncertain impaired group (95% confidence slope = 0.0046 to 0.0076 per month) declines nearly four times faster than the uncertain healthy group (95% confidence slope = 0.0011 to 0.0020 per month), while it shows separately different prognosis in the long term.

### Two Different Metabolism Patterns of Uncertain Cases

**FIGURE 3:**
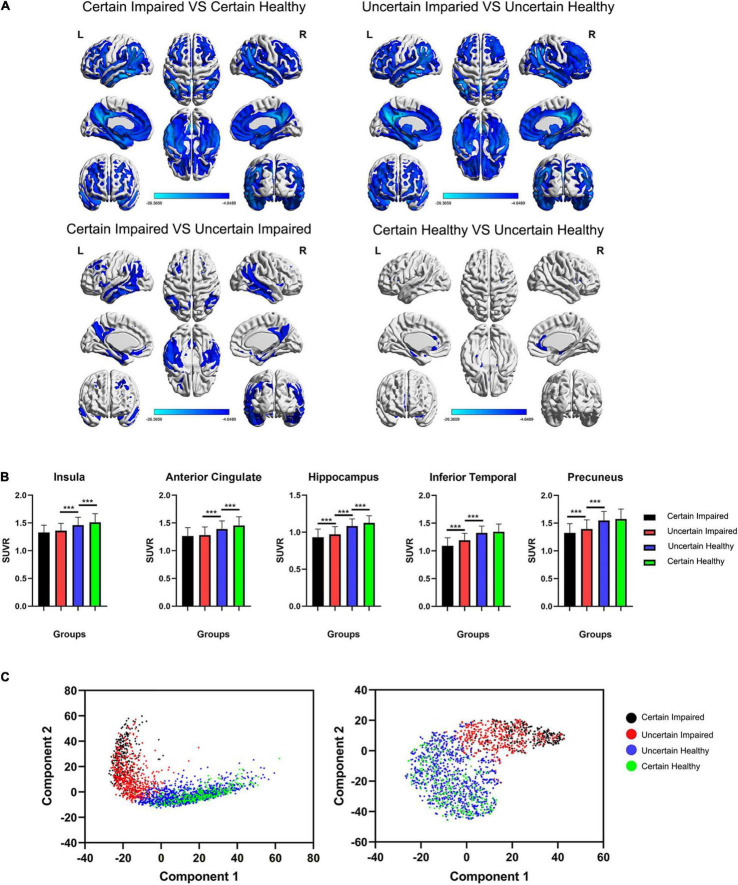
**(A)** The *t*-test maps of FDG-PET SUVr between four groups are shown among cerebrum, and slice views are found in Supplementary Figure 2. Only results of FWE corrected *p* < 0.05 were shown. The color bar represents the *t* value. **(B)** Comparison of the SUVr value of four groups in five typical ROIs. It is worth noting that the insula and anterior cingulate are significantly different between healthy groups, and the inferior temporal lobe and precuneus are significantly different between impaired groups. ***p < 0.001, FWE-corrected. **(C)** Principal component analysis (PCA) and *t*-distributed stochastic neighbor embedding (t-SNE) clustering for four groups of last flatten layer in the DL framework, showing separate distributions between diagnosed impaired and healthy cases using the DL framework. SUVr, standard uptake value ratio.

Owing to the DL diagnoses for uncertain cases, we could then manage to analyze the glucose metabolism state between four groups based on the labels, namely, certain/uncertain and impaired/healthy. The *t-*test results are shown in Figure 3A, and the *t*-test map view in slices is found in Supplementary Figure 2. In the first row, hypometabolism between impaired and healthy cases covers major regions among the cerebrum, including typical temporoparietal lobe and posterior cingulate, frontal lobe, limbic system, and subcutaneous nuclei, with less region of hypermetabolism in cerebellum regions 4 and 5.

However, in the second row of Figure 3A, two different metabolism patterns are associated with uncertain cases. For certain impaired vs. uncertain impaired cases, the hypometabolism concentrates on the binary medial frontal orbital cortex, temporoparietal lobe, hippocampus, parahippocampus, precuneus, and angular and middle posterior cingulate. For certain healthy vs. uncertain healthy cases, the hypometabolism concentrates only on the binary medial frontal orbital lobe, anterior cingulate, insula, hippocampus, and parahippocampus, with hypermetabolism in right cerebellum crus1 and cerebellum region 6. The significant differences in ROI are also associated with these different patterns (Figure 3B).

The *t*-SNE unsupervised topological representation of high-dimensional features extracted using the DL framework is shown in Figure 3C. It is worth noting that four groups showed continuous feature states from the order of certain impaired, uncertain impaired, uncertain healthy, and certain healthy, with clearly separate distributions between uncertain impaired and uncertain healthy cases. More importantly, the overlap between impaired cases was less than the overlap between healthy cases, while the areas of certain and uncertain healthy cases were much similar, meaning more diverse high-dimensional features of FDG-PET existed between certain and uncertain impaired cases.

### Mental State Features in Uncertain Cases

**FIGURE 4:**
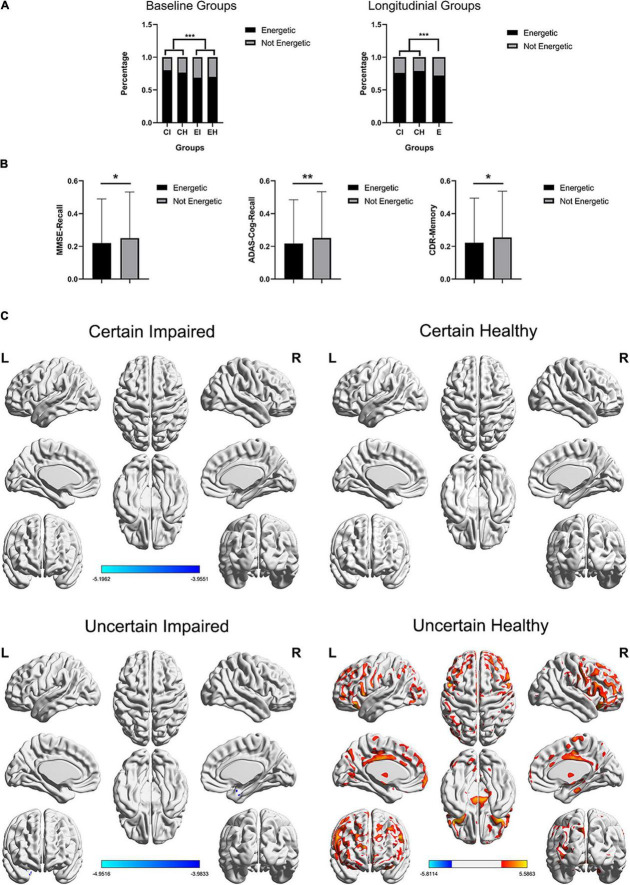
**(A)** Distribution of GDS-energy states. Baseline data include 2,386 cases and longitudinal data include 6,912 cases who completed all three neuropsychological tests, and the longitudinal uncertain group was not further diagnosed using the DL framework. **(B)** The SD of longitudinal tests within-subjects, including three neuropsychological tests, non-energetic subjects showed significant unstable test scores. ***p < 0.001, **p < 0.01, *p < 0.05. **(C)** The *t*-test maps between FDG-PET SUVr of energetic and non-energetic subjects, FDG corrected *p* < 0.05, respectively, in four groups, and slice views are found in Supplementary Figure 3. The color bar represents the *t* value.

To explore the latent relationships and interactions between mental state and uncertain cases, the chi-squared test for every single question of GDS and two-way ANOVA was applied on the total GDS score, with two factors (impaired/healthy and certain/uncertain cases) evaluated. *P*-value was corrected using FWE, resulting in *p* < 0.0031 for significance. As a result, only the GDS-Energy test was not significantly (*p* = 0.4329) correlated to the impaired/healthy memory impairment of subjects, but significantly (*p* < 0.001) correlated to the certain/uncertain state of subjects (Table 3), which means it influences the cases to be uncertain but did not influence the memory function. For GDS-Energy test, it asks “Do you feel full of energy?” toward the subjects, and the proportions that choose “yes” are 80.27% in certain impaired group, 68.80% in uncertain impaired group, 69.90% in the uncertain healthy group, and 76.44% in the certain healthy group. Uncertain groups are subjectively significantly less energetic than certain groups (*p* < 0.001), both in baseline and longitudinal studies (Figure 4A). Moreover, the subjects who were not energetic during the baseline showed more (*p* < 0.01, *p* < 0.05) unstable neuropsychological test results longitudinally than energetic subjects, including all three tests (Figure 4B). Additionally, the GDS-Memory score (*p* < 0.001) representing the self-assessment of memory capacity had both significant effects between impaired/healthy and certain/uncertain cases.

**TABLE 3:** *P*-value of chi-squared test for GDS single test and two-way ANOVA for GDS total scores, where impaired/healthy and certain/uncertain are two factors to be analyzed.

	Impaired/Healthy *p*-value	Certain/Uncertain *p*-value
GDS-satisfy	0.9599	0.1817
GDS-drop	**<0.001**	0.2798
GDS-empty	0.0527	0.9628
GDS-bored	0.0121	0.2560
GDS-spirit	0.1219	0.3865
GDS-afraid	0.4312	0.0720
GDS-happy	0.4199	0.6918
GDS-help	**<0.001**	0.7880
GDS-home	0.3250	0.0254
**GDS-memory^#^**	**<0.001**	**<0.001**
GDS-alive	0.1439	0.0975
GDS-worth	0.0174	0.4279
**GDS-energy^#^**	0.4329	**<0.001**
GDS-hope	**<0.001**	0.0199
GDS-better	0.1050	0.4254
**GDS-total^#^**	**<0.001**	0.0187

*Detailed questions of GDS are presented in Supplementary Table 1.*

*^#^Only these two points were significantly associated with certain/uncertain factors of all cases, after correcting the p-value threshold to 0.0031 by FWE. GDS, global deterioration scale.*

*Bold values were used to highlight the GDS scores that had significant association with both factors.*

### Energetic Mental State Mainly Affects Healthy Uncertain Cases

The influence of the energetic state was also evaluated using the glucose metabolism of FDG-PET. We applied *t*-tests between energetic and non-energetic cases, respectively, on the four diagnosing groups, and the *t*-test map view in slices is found in Supplementary Figure 3, which covers more concrete regions (Figure 4C). As a result, only uncertain healthy groups showed significant difference (*p* < 0.05; FDR-corrected), and the glucose metabolism of energetic subjects are stronger in the binary anterior middle cingulate, the wide range of frontal lobe, and a small region of the temporoparietal lobe, while weaker in binary cerebellum regions 8 and 9, which is partly similar to the significant difference between uncertain group and certain healthy group presented in Figure 3A.

## Discussion

As the results of neuropsychological tests might be conflicting within the same subject and lead to an uncertain case and diagnosis, we proposed a 3D-CNN-DL framework to diagnose memory impairment in uncertain cases using FDG-PET images, and the corresponding longitudinal study was proved to be clinically valid between positive and negative diagnoses. Then, by analyzing the FDG-PET and GDS between groups, we figured out that a mild-extent AD-related neurodegeneration state is a potential cause for an impaired sample to be uncertain, and a non-energetic mental state with a depression-related metabolism pattern is a potential cause for a healthy sample to be uncertain.

Neuropsychological tests, such as MMSE, ADAS-Cog, and CDR, are convenient and effective methods for screening and diagnosing dementia. These tests contain several questions for multiple cognitive aspects, including memory, orientation, attention, and language. Among these, memory impairment is the most common and vulnerable cognitive aspect during neurodegenerative diseases such as MCI (Petersen, 2004) and AD (Perry et al., 2000; Scheltens et al., 2016), so we focused on the memory aspect in this study. Another reason for choosing memory is the same test method in MMSE and ADAS-Cog, which both require subjects to recall given words that were learned before. By controlling the same method and adding the comprehensive assessment of CDR, the conflict results between them may not be blamed on the different designs or sensitivities of tests, but more on the mental and cognitive state of the subject being tested. As a result, up to 72.97% (1,741 out of 2,386) samples do not have consistent results between tests and are grouped into uncertain cases while using only three tests results as classification inputs showed poor diagnose validity, and also cannot reach the diagnosing capacity that the DL framework has achieved (Supplementary Table 2). This proved the necessity of data fusion (Zhang et al., 2020; Wang et al., 2021) between neuropsychological tests and neuroimages, such as FDG-PET or MRI, to certainly diagnose AD-related neurodegeneration (McKhann et al., 2011; Zhang et al., 2018).

Because of these disadvantages of neuropsychological tests, using the DL algorithm on neuroimages to diagnose neurodegenerative diseases is getting popular recently, especially in classifying AD dementia (Suk and Shen, 2013; Suk et al., 2014, 2015; Liu et al., 2015; Shi et al., 2018). The capability of 3D CNN allows integral input of the whole image information and extracts features from lower dimension to higher abstract dimension, with no human-designed *a priori* knowledge like the definition of ROI. To the best of our knowledge, most of these studies focused on training and applying the DL frameworks both on labeled cases (positive or negative pre-diagnosed by experts), and neglected to exploit the capability of diagnosing uncertain and difficult cases even for experts. So, we tried to use the FDG-PET images to diagnose uncertain memory impairment. To give an evaluation criterion for these diagnosing results, we studied all the available longitudinal progress (40 months on average) of 2,386 cases up to April 2020 in the ADNI dataset, which are more than 6,912 cases in the longitudinal study, then we found that the impaired diagnoses using the DL framework were significantly worse in longitudinal memory function decline than the healthy diagnoses using the DL framework. Especially in the CDR-Memory progress per month, the increasing slope of uncertain healthy cases was as flat as certain healthy cases, while the uncertain impaired cases showed around four times the increasing speed of healthy cases. This significant evidence proved that the DL framework could manage to tell apart the impaired and healthy impairment in uncertain cases and had clinical validity and application potential. Its clinical potential can be concluded as a more accurate diagnosis when facing conflicting neuropsychological test results and ensure less occurrence of misdiagnosis. Subsequently, its diagnosis for uncertain cases can reduce the potentially inappropriate medication and plan a more valid treatment in time, which is valuable for AD and MCI subjects.

Until present, the causes of conflict test conclusion and uncertain cases remain unclear. Major viewpoints blamed it on the different sensitivity or different design of tests (Perneczky et al., 2006). However, it lacks concrete evidence and specific research. So, this study gives a concrete explanation and evidence that both AD-related and depression-related causes can potentially lead to uncertain cases in different situations.

For AD-related causes, the FDG-PET *t*-test between groups with strict significance threshold and the *t*-SNE of feature in DL showed that the glucose metabolism intensity is decreasing progressively by this order of groups: certain healthy, uncertain healthy, uncertain impaired, and certain impaired. This evidence proves that the uncertain cases have a detectable neuropathological basis and shows an intermediate state between impaired and healthy cases. In other words, the neurodegenerative progress of an uncertain group is a state between healthy and diseased. The hypometabolism regions between certain and uncertain impaired cases are the typically affected regions of AD: frontal lobe, temporoparietal lobe, limbic systems such as the hippocampus and subcutaneous nuclei, which implies that the neurodegenerative extent of this group is not enough to reach a certain diagnosis but has the same impaired pattern.

For depression-related causes, we collected the GDS scores of each sample in the baseline, which contained 15 questions for different types of depression mental state. The chi-squared test showed two valuable results. First, the GDS-energy is significantly different (*p* < 0.001) in uncertain cases than certain cases but not different (*p* = 0.4329) between impaired and healthy cases. This means a non-energetic mental state is a key factor that can lead to uncertain cases regardless of the state of memory impairment. The baseline (Figure 4A) and longitudinal progress (Figure 4B) between energetic and non-energetic also verified that it influences the stability of test results. Second, GDS-memory is the self-assessed memory state of a subject, which is both significant between two factors of uncertain and certain cases, and impaired and healthy cases. This significance between the four groups showed that uncertain cases are not only caused by mental state but also correlated to clinical impairment such as memory. Moreover, the hypometabolism regions between certain and uncertain healthy cases are mainly binary medial frontal orbital lobe, anterior cingulate, insula, hippocampus and parahippocampus, with hypermetabolism in the cerebellum. These regions are not the same as the AD pattern but belong to a typical depression-related neuro circuit that has been widely studied (Mayberg et al., 1999, 2000; Phan et al., 2002; Phillips et al., 2003; Critchley, 2005). Correspondingly, the FDG-PET *t*-tests between energetic and non-energetic subjects in the four groups (Figure 4C) are only significant in uncertain healthy groups, while regions are mostly similar to this typical depression-related neuron circuit. This implies that the uncertainty in the healthy group might be affected by the non-energetic mental state. Although using different types of data, this conclusion supported the significant impact of depression in potential misdiagnoses. This result enlightens AD research field to focus more on the mental state such as depression (Hejl et al., 2002; Pier et al., 2012) of mild or suspected subjects, not only because it may confound the diagnosis, but also it has been identified as a risk factor for the cognitive decline (Diniz et al., 2013; Gimson et al., 2018; Marchant et al., 2020).

To conclude the relationship between the two causes, first, they are not independent or exclusive, but both exist and interact with each other by neurological basis such as neurodegeneration. Second, the priority of them varies when the subjects are healthy or impaired, while a mild-extent AD-related neurodegenerative progress is potentially the major cause of uncertain impaired cases, and non-energetic depression-related mental state is potentially the major cause of uncertain healthy cases, which could guide clinical practice to deal with uncertain cases reasonably and effectively. Third, the evidence of neurodegeneration and mental causes can verify each other, as proposed above.

Our study had several limitations. First, because we obtained samples from ADNI as a large multisite dataset and collected all available data to keep a large sample amount, the multisite effect of PET scanning and several unbalanced demographical information cannot be avoided, but we strictly used them as covariates in statistical tests. Second, although using MRI for PET preprocessing, because the scanning time interval between PET and MRI varies, we only chose FDG-PET as an evaluation of neurodegenerative progress, which might miss information that other modalities provided. Third, because of the priority of memory impairment in AD, we only focused on this aspect among many cognitive aspects, other aspects such as orientation and language or even total score are also valuable to be analyzed later.

## Conclusion

We proposed the DL framework based on FDG-PET for diagnosing uncertain cases of memory impairment related to AD, which was clinically reliable for diagnosing uncertain cases and proved valid in the corresponding longitudinal study. As for the cause and evidence of uncertain cases, for uncertain memory-impaired subjects, the uncertainty is mainly explained by mild-extent AD-related neurodegeneration. For uncertain memory-healthy subjects, the uncertainty is mainly explained by a non-energetic mental state and depression-related metabolism pattern.

## Data Availability Statement

Publicly available datasets were analyzed in this study. This data can be found here: http://adni.loni.usc.edu/. Please note that access is contingent on adherence to the ADNI Data Use Agreement and the publications’ policies.

## Author Contributions

WZ: methodology, software, validation, formal analysis, writing–original draft, writing–review and editing, and data curation. TZ: resources. TP: visualization. SZ: investigation. BN and HL: conceptualization and supervision. BS: supervision and project administration. All authors contributed to the article and approved the submitted version.

## Conflict of Interest

The authors declare that the research was conducted in the absence of any commercial or financial relationships that could be construed as a potential conflict of interest.

## Publisher’s Note

All claims expressed in this article are solely those of the authors and do not necessarily represent those of their affiliated organizations, or those of the publisher, the editors and the reviewers. Any product that may be evaluated in this article, or claim that may be made by its manufacturer, is not guaranteed or endorsed by the publisher.

## Footnotes

1
http://adni.loni.usc.edu/

2
www.fil.ion.ucl.ac.uk/spm

3
https://keras.io/

4
https://tensorflow.google.cn/

5
https://www.nvidia.cn/geforce/20-series/

6
https://www.csie.ntu.edu.tw/~cjlin/libsvm/

7
https://www.mathworks.com/

## References

[B1] Ashburner J. Friston K. J. (2005). Unified segmentation. *Neuroimage* 26 839–851. 10.1016/j.neuroimage.2005.02.018 15955494

[B2] Bergeron D. Flynn K. Verret L. Poulin S. Bouchard R. W. Bocti C. (2017). Multicenter Validation of an MMSE-MoCA conversion table. *J. Am. Geriatr. Soc.* 65 1067–1072. 10.1111/jgs.14779 28205215

[B3] Chang C. C. Lin C. J. (2011). LIBSVM: a library for support vector machines. *ACM Trans. Intell. Syst. Technol.* 2:27. 10.1145/1961189.1961199

[B4] Creavin S. T. Wisniewski S. Noel-Storr A. H. Trevelyan C. M. Hampton T. Rayment D. (2016). Mini-Mental State Examination (MMSE) for the detection of dementia in clinically unevaluated people aged 65 and over in community and primary care populations. *Cochrane Database Syst. Rev.* 2016:185. 10.1002/14651858.CD011145.pub2 PMC8812342 26760674

[B5] Critchley H. D. (2005). Neural mechanisms of autonomic, affective, and cognitive integration. *J. Comp. Neurol.* 493 154–166. 10.1002/cne.20749 16254997

[B6] De Jager C. A. Milwain E. Budge M. (2002). Early detection of isolated memory deficits in the elderly: the need for more sensitive neuropsychological tests. *Psychol. Med.* 32 483–491. 10.1017/S003329170200524X 11989993

[B7] Diniz B. S. Butters M. A. Albert S. M. Dew M. A. Reynolds C. F. III (2013). Late-life depression and risk of vascular dementia and Alzheimer’s disease: systematic review and meta-analysis of community-based cohort studies. *Br. J. Psychiatry* 202 329–335. 10.1192/bjp.bp.112.118307 23637108 PMC3640214

[B8] Du T. Bourdev L. Fergus R. Torresani L. Paluri M. (eds) (2015). “Learning spatiotemporal features with 3D convolutional networks,” in *Proceedings of the 2015 IEEE International Conference on Computer Vision; 2015 Dec 11-18*, (Santiago: IEEE).

[B9] Folstein M. F. Folstein S. E. McHugh P. R. (1975). Mini-mental state - practical method for granding cognitive state of patients for clinician. *J. Psychiatr. Res.* 12 189–198. 10.1016/0022-3956(75)90026-6 1202204

[B10] Gaugler J. E. Ascher-Svanum H. Roth D. L. Fafowora T. Siderowf A. Beach T. G. (2013). Characteristics of patients misdiagnosed with Alzheimer’s disease and their medication use: an analysis of the NACC-UDS database. *BMC Geriatr.* 13:137. 10.1186/1471-2318-13-137 24354549 PMC3878261

[B11] Gauthier S. Reisberg B. Zaudig M. Petersen R. C. Ritchie K. Broich K. (2006). Mild cognitive impairment. *Lancet* 367 1262–1270. 10.1016/S0140-6736(06)68542-5 16631882

[B12] Gimson A. Schlosser M. Huntley J. D. Marchant N. L. (2018). Support for midlife anxiety diagnosis as an independent risk factor for dementia: a systematic review. *BMJ Open* 8:e019399. 10.1136/bmjopen-2017-019399 29712690 PMC5969723

[B13] Hara K. Kataoka H. Satoh Y. (eds) (2017). “Learning spatio-temporal features with 3D residual networks for action recognition,” in *Proceedings of the 16th IEEE International Conference on Computer Vision (ICCV); 2017 Oct 22-29*, (Venice). 10.1109/ICCVW.2017.373

[B14] Hejl A. Hogh P. Waldemar G. (2002). Potentially reversible conditions in 1000 consecutive memory clinic patients. *J. Neurol. Neurosurg. Psychiatry* 73 390–394. 10.1136/jnnp.73.4.390 12235305 PMC1738080

[B15] Hosokawa C. Ishii K. Kimura Y. Hyodo T. Hosono M. Sakaguchi K. (2015). Performance of C-11-Pittsburgh compound B PET binding potential images in the detection of amyloid deposits on equivocal static images. *J. Nucl. Med.* 56 1910–1915. 10.2967/jnumed.115.156414 26359262

[B16] Jack C. R. Jr. Bennett D. A. Blennow K. Carrillo M. C. Dunn B. Haeberlein S. B. (2018). NIA-AA research framework: toward a biological definition of alzheimer’s disease. *Alzheimers Demen.* 14 535–562. 10.1016/j.jalz.2018.02.018 29653606 PMC5958625

[B17] Jack C. R. Jr. Bernstein M. A. Fox N. C. Thompson P. Alexander G. Harvey D. (2008). The Alzheimer’s Disease Neuroimaging Initiative (ADNI): MRI methods. *J. Magn. Reson. Imaging* 27 685–691. 10.1002/jmri.21049 18302232 PMC2544629

[B18] Jagust W. J. Bandy D. Chen K. Foster N. L. Landau S. M. Mathis C. A. (2010). The alzheimer’s disease neuroimaging initiative positron emission tomography core. *Alzheimers Demen.* 6 221–229. 10.1016/j.jalz.2010.03.003 20451870 PMC2920531

[B19] Jiang Y. Yang H. Zhao J. Wu Y. Zhou X. Cheng Z. (2020). Reliability and concurrent validity of Alzheimer’s disease assessment scale-cognitive subscale, Chinese version (ADAS-Cog-C) among Chinese community-dwelling older people population. *Clin. Neuropsychol.* 34(Suppl. 1), 43–53. 10.1080/13854046.2020.1750704 32279575

[B20] Kingma D. B. J. (2015). *Adam: A Method for Stochastic Optimization. ICLR.* arXiv:1412.6980. Available online at: https://arxiv.org/abs/1312.4400

[B21] Klunk W. E. Koeppe R. A. Price J. C. Benzinger T. L. Devous Sr M. D. Jagust W. J. (2015). The centiloid project: standardizing quantitative amyloid plaque estimation by PET. *Alzheimers Demen.* 11 1-15.e1-4. 10.1016/j.jalz.2014.07.003 25443857 PMC4300247

[B22] LeCun Y. Bengio Y. Hinton G. (2015). Deep learning. *Nature* 521 436–444. 10.1038/nature14539 26017442

[B23] Lin M. Chen Q. Yan S. (2015). “Network in network,” *ICLR*. Available online at: https://arxiv.org/abs/1312.4400

[B24] Liu S. Liu S. Cai W. Che H. Pujol S. Kikinis R. (2015). Multimodal neuroimaging feature learning for multiclass diagnosis of alzheimer’s disease. *IEEE Trans. Biomed. Eng.* 62 1132–1140. 10.1109/TBME.2014.2372011 25423647 PMC4394860

[B25] Marchant N. L. Lovland L. R. Jones R. Pichet Binette A. Gonneaud J. Arenaza-Urquijo E. M. (2020). Repetitive negative thinking is associated with amyloid, tau, and cognitive decline. *Alzheimers Dement.* 16 1054–1064. 10.1002/alz.12116 32508019

[B26] Matthews F. E. Stephan B. C. M. McKeith I. G. Bond J. Brayne C., and Medical Research Council Cognitive Function and Ageing Study (2008). Two-year progression from mild cognitive impairment to dementia: to what extent do different definitions agree? *J. Am. Geriatr. Soc.* 56 1424–1433. 10.1111/j.1532-5415.2008.01820.x 18662209

[B27] Mayberg H. S. Brannan S. K. Tekell J. L. Silva J. A. Mahurin R. K. McGinnis S. (2000). Regional metabolic effects of fluoxetine in major depression: serial changes and relationship to clinical response. *Biol. Psychiatry* 48 830–843. 10.1016/S0006-3223(00)01036-2 11063978

[B28] Mayberg H. S. Liotti M. Brannan S. K. McGinnis S. Mahurin R. K. Jerabek P. A. (1999). Reciprocal limbic-cortical function and negative mood: converging PET findings in depression and normal sadness. *Am. J. Psychiatry* 156 675–682. 10327898 10.1176/ajp.156.5.675

[B29] McKhann G. M. Knopman D. S. Chertkow H. Hyman B. T. Jack C. R. Jr. Kawas C. H. (2011). The diagnosis of dementia due to Alzheimer’s disease: recommendations from the National Institute on Aging-Alzheimer’s Association workgroups on diagnostic guidelines for Alzheimer’s disease. *Alzheimers Demen.* 7 263–269. 10.1016/j.jalz.2011.03.005 21514250 PMC3312024

[B30] Mitchell A. J. (2009). A meta-analysis of the accuracy of the mini-mental state examination in the detection of dementia and mild cognitive impairment. *J. Psychiatr. Res.* 43 411–431. 10.1016/j.jpsychires.2008.04.014 18579155

[B31] Mohs R. C. Rosen W. G. Davis K. L. (1983). The Alzheimer’s disease assessment scale: an instrument for assessing treatment efficacy. *Psychopharmacol. Bull.* 19:448. 6635122

[B32] Morris J. C. (1993). The Clinical Dementia Rating (CDR): current version and scoring rules. *Neurology* 43 2412–2414. 10.1212/WNL.43.11.2412-a 8232972

[B33] Mullergartner H. W. Links J. M. Prince J. L. Bryan R. N. McVeigh E. Leal J. P. (1992). Measurement of radiotracer concentration in brain gray-matter using positron emission tomography - mri-based correction for partial volume effects. *J. Cereb. Blood Flow Metab.* 12 571–583. 10.1038/jcbfm.1992.81 1618936

[B34] Ortiz A. Munilla J. Gorriz J. M. Ramirez J. (2016). Ensembles of deep learning architectures for the early diagnosis of the alzheimer’s disease. *Int. J. Neural Syst.* 26:23. 10.1142/S0129065716500258 27478060

[B35] Perneczky R. Wagenpfeil S. Komossa K. Grimmer T. Diehl J. Kurz A. (2006). Mapping scores onto stages: mini-mental state examination and clinical dementia rating. *Am. J. Geriatr. Psychiatry* 14 139–144. 10.1097/01.JGP.0000192478.82189.a8 16473978

[B36] Perry R. J. Watson P. Hodges J. R. (2000). The nature and staging of attention dysfunction in early (minimal and mild) Alzheimer’s disease: relationship to episodic and semantic memory impairment. *Neuropsychologia* 38 252–271. 10.1016/S0028-3932(99)00079-2 10678692

[B37] Petersen R. C. (2004). Mild cognitive impairment as a diagnostic entity. *J. Int. Med.* 256 183–194. 10.1111/j.1365-2796.2004.01388.x 15324362

[B38] Phan K. L. Wager T. Taylor S. F. Liberzon I. (2002). Functional neuroanatomy of emotion: a meta-analysis of emotion activation studies in PET and fMRI. *Neuroimage* 16 331–348. 10.1006/nimg.2002.1087 12030820

[B39] Phillips M. L. Drevets W. C. Rauch S. L. Lane R. (2003). Neurobiology of emotion perception II: implications for major psychiatric disorders. *Biol. Psychiatry* 54 515–528. 10.1016/S0006-3223(03)00171-9 12946880

[B40] Pier K. S. Briggs M. C. Pasculli R. M. Kellner C. H. (2012). Successful electroconvulsive therapy for major depression misdiagnosed as alzheimer dementia. *Am. J. Geriatr. Psychiatry* 20 909–910. 10.1097/JGP.0b013e318254619a 22771953 PMC3592561

[B41] Rikkert M. G. M. O. Tona K. D. Janssen L. Burns A. Lobo A. Robert P. (2011). Validity, reliability, and feasibility of clinical staging scales in dementia: a systematic review. *Am. J. Alzheimers Dis. Other Demen.* 26 357–365. 10.1177/1533317511418954 21914671 PMC10845587

[B42] Scheltens P. Blennow K. Breteler M. M. B. de Strooper B. Frisoni G. B. Salloway S. (2016). Alzheimer’s disease. *Lancet* 388 505–517. 10.1016/S0140-6736(15)01124-1 26921134

[B43] Shen D. Wu G. Suk H.-I. (2017). “Deep learning in medical image analysis,” in *Annual Review of Biomedical Engineering*, Vol. 19 ed. Yarmush M. L. (Palo Alto, CA: Annual Review Inc), 221–248. 10.1146/annurev-bioeng-071516-044442 PMC5479722 28301734

[B44] Shen T. Jiang J. Lu J. Wang M. Zuo C. Yu Z. (2019). Predicting alzheimer disease from mild cognitive impairment with a deep belief network based on 18F-FDG-PET images. *Mol. Imaging* 18:1536012119877285. 10.1177/1536012119877285 31552787 PMC6764042

[B45] Shi J. Zheng X. Li Y. Zhang Q. Ying S. (2018). Multimodal neuroimaging feature learning with multimodal stacked deep polynomial networks for diagnosis of alzheimer’s disease. *IEEE J. Biomed. Health Inf.* 22 173–183. 10.1109/JBHI.2017.2655720 28113353

[B46] Shim Y. S. Roe C. M. Buckles V. D. Morris J. C. (2013). Clinicopathologic study of alzheimer’s disease: alzheimer mimics. *J. Alzheimers Dis.* 35 799–811. 10.3233/JAD-121594 23481687 PMC3725959

[B47] Son H. J. Oh J. S. Oh M. Kim S. J. Lee J. H. Roh J. H. (2020). The clinical feasibility of deep learning-based classification of amyloid PET images in visually equivocal cases. *Eur. J. Nucl. Med. Mol. Imaging.* 47 332–341. 10.1007/s00259-019-04595-y 31811343

[B48] Spasov S. Passamonti L. Duggento A. Lio P. Toschi N., and Alzheimer’s Disease Neuroimaging Initiative (2019). A parameter-efficient deep learning approach to predict conversion from mild cognitive impairment to Alzheimer’s disease. *Neuroimage.* 189 276–287. 10.1016/j.neuroimage.2019.01.031 30654174

[B49] Spencer R. J. Wendell C. R. Giggey P. P. Katzel L. I. Lefkowitz D. M. Siegel E. L. (2013). Psychometric limitations of the mini-mental state examination among nondemented older adults: an evaluation of neurocognitive and magnetic resonance imaging correlates. *Exp. Aging Res.* 39 382–397. 10.1080/0361073X.2013.808109 23875837

[B50] Suk H.-I. Shen D. (2013). “Deep learning-based feature representation for AD/MCI classification,” in *16th International Conference on Medical Image Computing and Computer Assisted Intervention (MICCAI)*. 10.1007/978-3-642-40763-5_72 PMC4029347 24579188

[B51] Suk H.-I. Lee S.-W. Shen D., and Alzheimer’s Disease Neuroimaging Initiative (2014). Hierarchical feature representation and multimodal fusion with deep learning for AD/MCI diagnosis. *Neuroimage* 101 569–582. 10.1016/j.neuroimage.2014.06.077 25042445 PMC4165842

[B52] Suk H.-I. Lee S.-W. Shen D., and Alzheimer’s Disease Neuroimaging Initiative (2015). Latent feature representation with stacked auto-encoder for AD/MCI diagnosis. *Brain Struct. Funct.* 220 841–859. 10.1007/s00429-013-0687-3 24363140 PMC4065852

[B53] Trzepacz P. T. Hochstetler H. Wang S. Walker B. Saykin A. J., and Alzheimer’s Disease Neuroimaging Initiative (2015). Relationship between the montreal cognitive assessment and mini-mental state examination for assessment of mild cognitive impairment in older adults. *BMC Geriatr.* 15:107. 10.1186/s12877-015-0103-3 26346644 PMC4562190

[B54] van der Maaten L. Hinton G. (2008). Visualizing data using t-SNE. *J. Mach. Learn. Res.* 9 2579–2605.

[B55] Wang S. Celebi M. E. Zhang Y.-D. Yu X. Lu S. Yao X. (2021). Advances in data preprocessing for biomedical data fusion: an overview of the methods, challenges, and prospects. *Inf. Fusion* 76 376–421. 10.1016/j.inffus.2021.07.001

[B56] Whitwell J. L. Graff-Radford J. Tosakulwong N. Weigand S. D. Machulda M. M. Senjem M. L. (2018). Imaging correlations of tau, amyloid, metabolism, and atrophy in typical and atypical Alzheimer’s disease. *Alzheimers Demen.* 14 1005–1014. 10.1016/j.jalz.2018.02.020 29605222 PMC6097955

[B57] Wilson R. S. Leurgans S. E. Boyle P. A. Bennett D. A. (2011). Cognitive decline in prodromal alzheimer disease and mild cognitive impairment. *Arch. Neurol.* 68 351–356. 10.1001/archneurol.2011.31 21403020 PMC3100533

[B58] Wind A. W. Schellevis F. G. Van Staveren G. Scholten R. J. P. M. Jonker C. Van Eijk J. T. M. (1997). Limitations of the mini-mental state examination in diagnosing dementia in general practice. *Int. J. Geriatr. Psychiatry.* 12 101–108. 10.1002/(SICI)1099-1166(199701)12:1<101::AID-GPS469>3.0.CO;2-R 9050431

[B59] Zhang Y. Wang S. Sui Y. Yang M. Liu B. Cheng H. (2018). Multivariate approach for alzheimer’s disease detection using stationary wavelet entropy and predator-prey particle swarm optimization. *J. Alzheimers Dis.* 65 855–869. 10.3233/JAD-170069 28731432

[B60] Zhang Y.-D. Dong Z. Wang S.-H. Yu X. Yao X. Zhou Q. (2020). Advances in multimodal data fusion in neuroimaging: overview, challenges, and novel orientation. *Inf. Fusion* 64 149–187. 10.1016/j.inffus.2020.07.006 32834795 PMC7366126

