# Supplementary material for: Deep Learning With : 18F-Fluorodeoxyglucose-PET Gives Valid Diagnoses for the Uncertain Cases in Memory Impairment of Alzheimer’s Disease
Source: Front Aging Neurosci. 2021 Dec 15;13:764272. doi: 10.3389/fnagi.2021.764272 (PMC8715958; doi:10.3389/fnagi.2021.764272)
Supplement: Supplementary file 1 [file Data_Sheet_1.PDF]

Supplement

1.GDS tests

Supplementary Table.1 Questions of GDS tests

Abbreviations	Questions
GDS-Satisfy	Are you basically satisfied with your life?
GDS-Drop	Have you dropped many of your activities and interests?
GDS-Empty	Do you feel that your life is empty?
GDS-Bored	Do you often get bored?
GDS-Spirit	Are you in good spirits most of the time?
GDS-Afraid	Are you afraid that something bad is going to happen to you?
GDS-Happy	Do you feel happy most of the time?
GDS-Help	Do you often feel helpless?
GDS-Home	Do you prefer to stay at home, rather than going out and doing things?
GDS-Memory	Do you feel that you have more problems with memory than most?
GDS-Alive	Do you think it is wonderful to be alive now?
GDS-Worth	Do you feel worthless the way you are now?
GDS-Energy	Do you feel full of energy?
GDS-Hope	Do you feel that your situation is hopeless?
GDS-Better	Do you think that most people are better off than you are?
GDS-Total	Total score

2.Other models and neuropsychological tests are insufficient for equivocal diagnoses

Supplementary Table.2 Classification consistency with DL of 6 models using test scores as inputs, regarding the diagnosing of deep learning model as standard, applied in equivocal set.

Test Scores as Input	Accuracy	Precision	Recall	F1 Score
C-SVM (linear kernel)	73.35%	57.21%	65.05%	60.88%
C-SVM (radial kernel)	73.00%	56.08%	70.62%	62.52%
Nu-SVM (linear kernel)	73.29%	58.33%	56.76%	57.53%
Nu-SVM (radial kernel)	72.95%	56.54%	65.41%	60.65%
Linear regression	72.83%	57.68%	55.50%	56.57%
logistic regression	73.46%	57.56%	63.78%	60.51%

Supplementary Table.3 Classification consistency with DL of 6 models using FDG PET as inputs, regarding the diagnosing of deep learning model as standard, applied in equivocal set.

FDG PET as Input	Accuracy	Precision	Recall	F1 Score
C-SVM (linear kernel)	86.10%	76.84%	80.72%	78.73%
C-SVM (radial kernel)	78.40%	63.10%	77.66%	69.63%
Nu-SVM (linear kernel)	81.28%	67.43%	79.82%	73.10%
Nu-SVM (radial kernel)	81.10%	67.23%	79.46%	72.83%
Linear regression	80.36%	71.01%	64.86%	67.80%

logistic regression	80.24%	70.89%	64.50%	67.55%

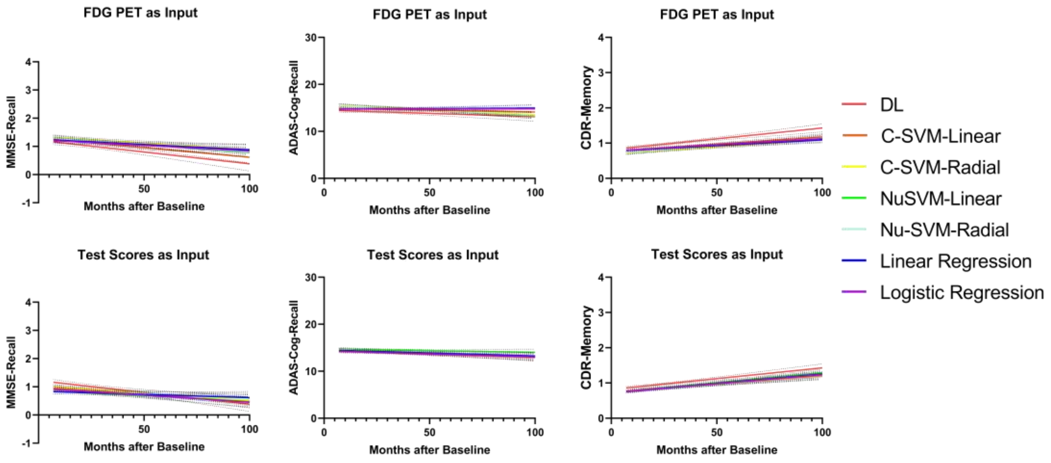

Supplement Fig.1 a: Longitudinal study of neuropsychological tests of positive diagnoses by 7 models, using baseline FDG-PET as input. b: Longitudinal study of neuropsychological tests of positive diagnoses by 7 models, using baseline neuropsychological tests as input except DL.

To evaluate whether the tests are sufficient for the diagnosing of equivocal cases, 6 other models were applied to diagnose equivocal cases using three test scores (MMSE-Recall, ADAS-Cog-Recall and CDR-Memory) or FDG PET as inputs. The performance compared to DL results was shown in the Supplementary Table.1 and Table.2.

The performance was also evaluated by longitudinal tracing, other 6 models comparing with DL was shown in Supplement Fig.1. In Fig.1a, 6 models used baseline SUVR value in ROIs of FDG-PET as input, and in Fig.1b, 6 models used baseline neuropsychological tests as input. It turned out that the impaired diagnosed by DL always showed the worst progress, so DL outperformed other models in all cases evaluated in our research.

3.Slice view of t-test map in Fig.3a

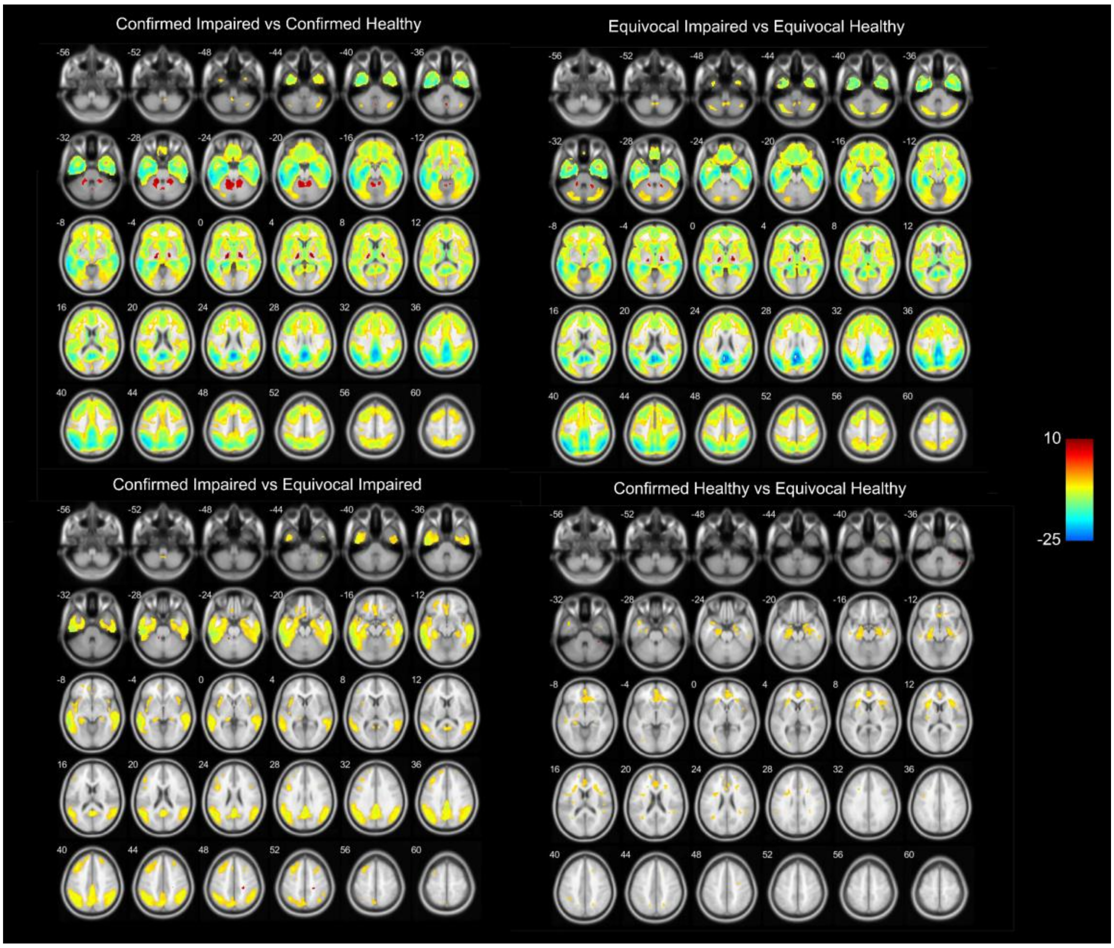

Supplement Fig.2 T-tests maps of FDG PET SUVr between four groups in the manuscript Fig.3a. p<0.05 FWE corrected, cluster size>5. Color bar represents the t value.

4.Slice view of t-test map in Fig.4c

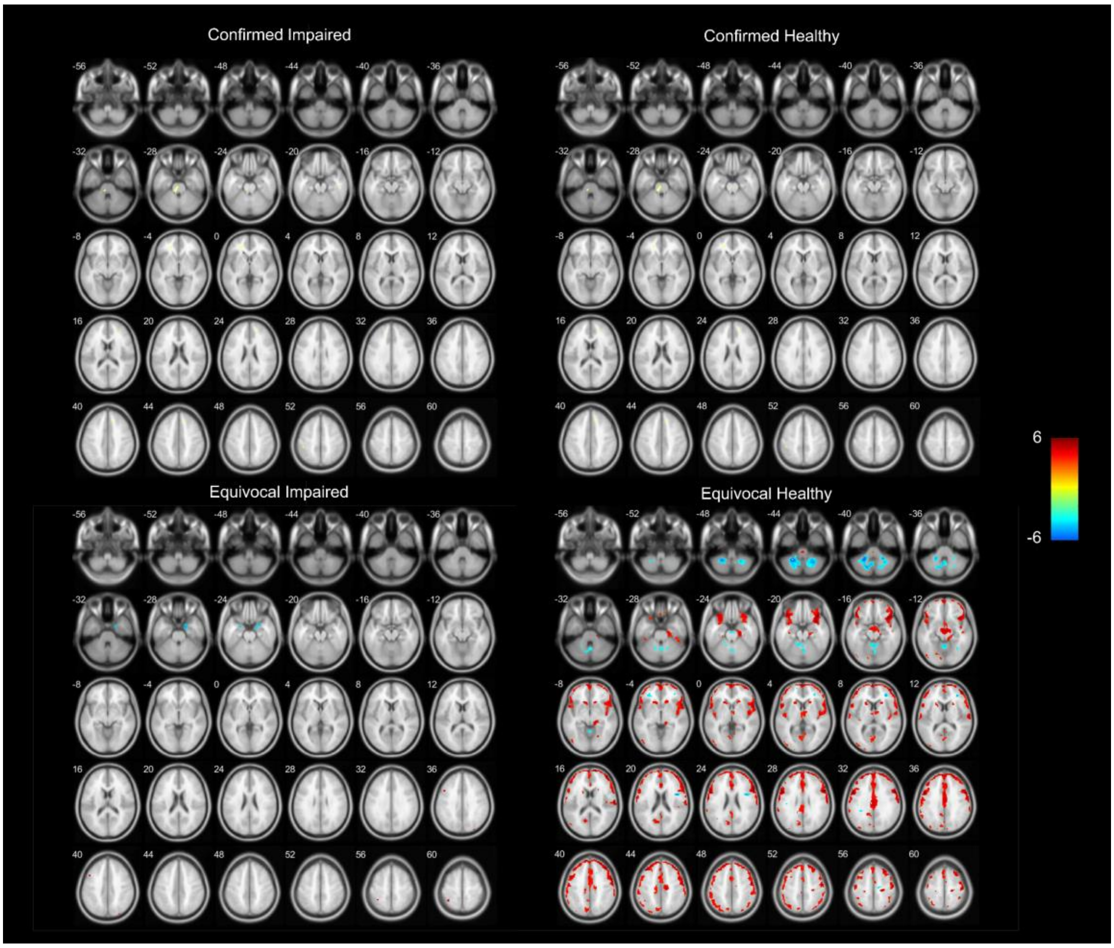

Supplement Fig.3 T-tests maps between FDG PET SUVr of energetic and not-energetic subjects in the manuscript Fig.4c, FDG corrected $p<0.05$, respectively in four groups. Color bar represents the t value.
